# High Intrathoracic Anastomosis with Thoracoscopy Is Safe and Feasible for Treatment of Esophageal Squamous Cell Carcinoma

**DOI:** 10.1371/journal.pone.0152151

**Published:** 2016-03-24

**Authors:** Hyun Woo Jeon, Jae Kil Park, Kyo Young Song, Sook Whan Sung

**Affiliations:** 1 Department of Thoracic and Cardiovascular Surgery, Seoul St. Mary’s Hospital, College of Medicine, The Catholic University of Korea, Seoul, Republic of Korea; 2 Department of Surgery, Seoul St. Mary’s Hospital, College of Medicine, The Catholic University of Korea, Seoul, Republic of Korea; Baylor College of Medicine, UNITED STATES

## Abstract

**Background:**

Minimally invasive esophagectomy (MIE) has the potential to reduce the morbidity and mortality of esophageal cancer surgery. Esophageal squamous cell carcinoma (ESCC) has a high incidence of earlier lymphatic spread and is usually located more proximal to the incisor than esophageal adenocarcinoma; consequently, the anastomosis should be made more proximal in the thorax or in the neck. We adopted the proximal intrathoracic anastomotic technique using thoracoscopy for mid-to-lower ESCC.

**Methods:**

From October 2010 to August 2014, fifty-eight consecutive patients underwent MIE for ESCC. After laparoscopic gastric tubing, thoracoscopic esophageal resection and reconstruction were performed using a 28-mm circular stapler following radical mediastinal lymph node dissection. We tried to make an anastomosis at the apex of the chest. Postoperative outcomes, including overall survival and recurrence, were assessed.

**Results:**

The mean patient age was 64.3±9 years. The mean operative time was 371.8±51.6 minutes, and the duration of the thorax procedure was 254.8±38.3 minutes. The mean number of lymph nodes dissected was 31±11.7. The mean intensive care unit (ICU) stay and hospital stay were 3.5±8.2 hours and 13.6±7.4 days, respectively. The level of anastomosis was 22.3±1.8cm from the incisor. One patient died of uncontrolled sepsis due to necrosis of the gastric graft. Two patients developed small contained leakage. Nine patients exhibited distant metastasis during the follow-up period.

**Conclusion:**

Thoracoscopic intrathoracic anastomosis at the proximal esophagus is feasible and safe.

## Introduction

Surgical resection is known to be curative for locoregional disease in the case of esophageal cancer. Open surgery is the current standard treatment; however, it is a complex procedure with high morbidity and mortality. To overcome this problem, minimally invasive esophagectomy (MIE) was introduced in the early 1990s and has yielded good postoperative results (morbidity: 4%; mortality: 1%) [1]. Unlike adenocarcinoma, ESCC is usually located higher, and regional lymphatic spread and satellite lesions are more common [2]. More proximal anastomosis is required to achieve negative proximal margins, which require either a cervical or high intrathoracic anastomosis. However, cervical anastomosis is preferred regardless of the tumor location in MIE. Generally, complications of anastomotic leakage, stricture, and nerve injury are more common with cervical anastomosis [3], and an additional neck incision is required. We have adopted the thoracoscopic high intrathoracic anastomosis for mid to lower ESCC and present our experience and outcomes.

## Materials and Methods

This study was a retrospective review conducted with Seoul St. Mary’s Hospital Institutional Review Board approval (KC 12RISI0848), and all patient information was anonymized and de-identified prior to analysis. Between October 2010 and August 2014, 139 patients underwent esophagectomy and esophageal reconstruction for esophageal cancer. Finally, we identified and reviewed the cases of 58 consecutive patients who underwent minimally invasive Ivor Lewis esophagectomy performed by a single surgeon. The operative indications were as follows: (a) mid-to-lower esophageal cancer (mean distance from the incisor, 30.9±4.1cm); (b) non-T4 esophageal cancer; and (c) no history of laparotomy or thoracotomy. If the cancer was clinical stage III, neoadjuvant treatment was conducted. In every patient, the laparoscopic gastric tubing procedure was performed first, and thoracoscopic esophagectomy and esophagogastrostomy were then performed using a 28-mm circular stapler. The anastomosis was carried out at the apex of the chest. Preoperative assessment included endoscopy, endoscopic ultrasonography (EUS), pulmonary function tests, echocardiography, chest computed tomography (CT), and positron emission tomography (PET) in every patient. The complications, mortality, and early outcomes after treatment were reviewed.

### Surgical technique

The patient was placed in the supine position, and a double-lumen endotracheal tube was placed. Five-port abdominal incisions were made for gastric mobilization. A 10.5-mm port was placed in the umbilicus for the insertion of a 30° angle scope. Two 5-mm ports were placed bilaterally in the subcostal region at the mid-clavicular region, and 12-mm ports were placed between these two 5-mm ports on both sides. The greater omentum was incised using the Harmonic scalpel (Ethicon Endo-Surgery, Inc., Cincinnati, Ohio, USA). Dissection was carried out along the greater curvature of the stomach from the insertion of the right gastroepiploic artery. The gastrosplenic ligament and short gastric vessels were divided and the lesser omentum was incised. The left gastric artery and vein were identified and divided using an endoclip. The gastrohepatic ligament was then divided. The esophagus was mobilized from the esophageal hiatus. Usually hiatus was enlarged a little bit with small division of right sided crura to avoid stomach compression at the hiatus postoperatively. Pyloromyotomy was performed. Partial gastric tubing procedure were performed using linear stapler at distal two third of stomach alongside lesser curvature with creation of 5 to 6 cm width of the stomach. Regional lymph nodes were dissected during the procedure without jejunostomy [4, 5].

**Fig 1:**
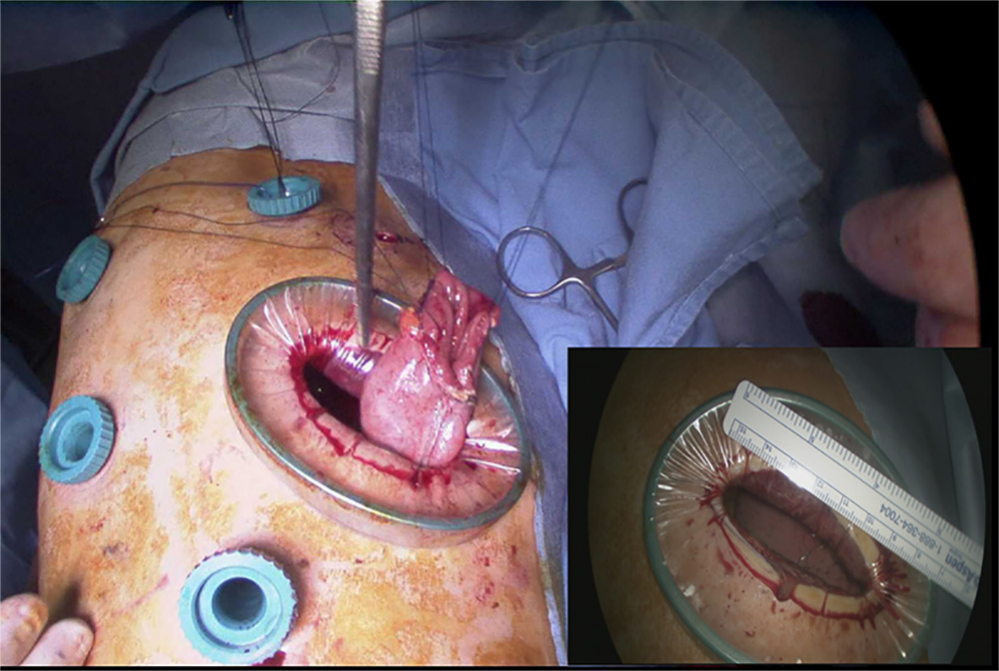
The port incision including a 6cm utility incision. A 10.5 mm port was placed in the mid-axillary line of the 7th intercostal space for insertion of the 30° camera. A utility incision was made at the anterior axillary line of the 5th or 6th intercostal space. A 10.5 mm port was placed below the scapular tip. A 10.5 mm port was placed at the anterior axillary line of the 3rd intercostal space.

**Fig 2:**
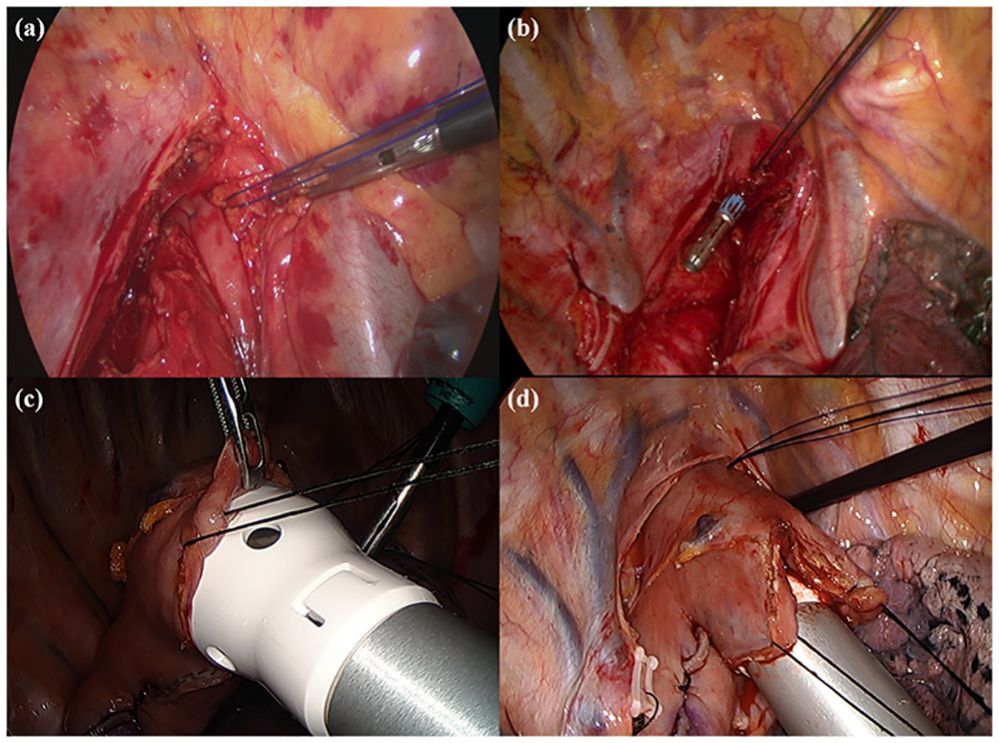
Anastomotic technique for the thoracoscopic Ivor Lewis procedure. (a) Single muscular purse string suture on the proximal esophagus. (b) The anvil of a 28-mm circular stapler was placed in the proximal esophagus. (c) EEA body insertion into the gastric conduit was performed inside the chest under thoracoscopy. (d) High intrathoracic anastomosis was carried out.

The patient’s position was changed to the left lateral decubitus, and a 6-cm utility incision with four ports was made in the right side of the chest (Fig 1). After selective single-lung ventilation, the azygos vein was divided using the endostapler (TriStapler; Covidien, Norwalk, CT). The mediastinal pleura was completely opened over the thoracic esophagus. Esophageal dissection was performed using ultrasonic scissors. During the esophageal dissection, regional lymph nodes and soft tissue were removed from the level of the thoracic inlet to the diaphragm, including recurrent laryngeal lymph nodes and subcarinal lymph nodes. The thoracic duct was routinely ligated. After the intrathoracic esophagus was mobilized, a manual purse-string suture of the muscular layer was placed at the highest level of the thoracic esophagus using 2–0 prolene^®^ (Fig 2a).

The esophagus was opened longitudinally 3 to 4 centimeters just below the purse-string suture. The anvil of a 28-mm circular stapler was carefully placed in the proximal esophagus through the esophageal opening, and the purse-string suture was tied around the central rod (Fig 2b). Additionally, the esophagus was divided just below the tied purse string suture. The stomach was pulled into the thorax through the esophageal hiatus. The dissected esophagus and stomach were pulled out through the utility incision, and a final gastric tubing procedure (>5cm in width of gastric conduit) was carried out using a linear stapler, leaving a 4-cm opening for subsequent EEA body insertion (DST EEA 28; Tyco, Healthcare, Norwalk, CT). The stomach graft was then returned to the thoracic cavity. The EEA body was inserted into the stomach graft (Fig 2c), and the spike penetrated the stomach wall. The female end of the anvil was joined to the male end of the body spike, and the device was fired to create the anastomosis (Fig 2d). After ensuring the internal mucosal integrity, the stomach opening was closed with a linear stapler. Proximal margins were checked by frozen sectioning. The gastric tube was positioned in the posterior mediastinum, and the incised pleura was closed with interrupted sutures including the stomach wall (stapling line) for the prevention of gastric elongation or bulging into the pleural cavity [6]. A single chest tube was placed and removed when the drainage was less than 200 ml/day.

## Results

The mean patient age was 64.3±9 years (Table 1), and fifty-four of the patients were men (93.1%). Eight patients (13.8%) received neoadjuvant chemoradiation treatment for clinically advanced esophageal cancer (stage III). The tumor was located near the tracheal bifurcation or lower thoracic esophagus, and all of the cases were ESCC (mean 30.9cm from incisor). The operative time and thoracoscopic time were 371.8±51.6 minutes and 254.8±38.3 minutes, respectively, and there was no case that was converted to the open procedure or McKeown operation. Esophageal reconstruction was conducted using a 28-mm circular stapler for all patients. Two patients (3.4%) underwent concomitant operations. Right upper lobe pulmonary lobectomy and cholecystectomy were performed for the primary lung cancer and gall bladder stone, respectively. Complete resection was achieved in every patient. The mean length of the proximal resection margin was 5.2±2.3 cm. Satellite lesions that were not identified on preoperative endoscopy or imaging studies were identified in 4 patients by the final pathologic study, and the distance from the satellite tumor ranged from 1.5 to 2.5 cm in specimens. Pathologically, there were 28 stage I, 19 stage II, and 11 stage III esophageal cancers. The number of dissected lymph nodes was 31±11.7 (Table 2). The mean tumor size was 2.8±1.6 cm. The mean intensive care unit duration was 3.5±8.2 hours, and the mean hospital duration was 13.6±7.4 days. Diet was resumed and advanced from postoperative days 5 to 8 depending on the patients’ physical signs, laboratory findings, and general condition. The barium swallow test was not carried out unless anastomotic leakage was suspected.

**Table 1:** Baseline Patient Characteristics.

Characteristic	Total (n = 58)
	Mean±SD (cm) or n (%)
Age	64.3±9
Gender	Male: 54(93.1)
HBP	24 (41.4)
Current smoker	17 (29.3)
Tumor location	30.9±4.1
Clinical stage	
Stage I	31 (53.4)
Stage II	18 (31.0)
Stage III	9 (15.5)

Data were presented as the mean±SD or frequencies and percentages as appropriate

HBP: Hypertension

**Table 2:** Operative data.

Variable	Value
	Mean±SD or n(%)
Operative time (minutes)	
Total surgery time	371.8±51.6
Thoracoscopic time	254.8±38.3
R0 resection (%)	58 (100)
Conversion to thoracotomy (%)	None
Length of proximal resection margin (cm)	5.2±2.3
Number of lymph node dissected	31±11.7
Tumor size (cm)	2.8±1.6
pStage	
p/yp Stage I	26/2 (48.3)
p/yp Stage II	16/3 (32.8)
p/yp Stage III	8/3 (18.9)
ICU stay (hours)	3.5±8.2
Hospital stay (days)	13.6±7.4
The level of anastomosis from incisor (cm)	22.3±1.8

Data were presented as the mean±SD or frequencies and percentages as appropriate

R0 resection: complete resection without residual tumor, p stage: Pathologic stage, yp stage: pathologic stage after neoadjuvant treatment. ICU:Intensive care unit.

### Complications

Two patients received transfusion (3.4%). One patient received an intraoperative transfusion due to a spleen laceration during the division of the gastrosplenic ligament. Another patient required a postoperative transfusion because of bleeding from the anastomosis on postoperative day 2.

Mortality occurred in one patient (1.8%, Table 3). This patient developed proximal gastric conduit necrosis that may be attributable to a narrowly designed gastric conduit. Reoperation was carried out. However, the patient died of sepsis on postoperative day 18. Two patients developed a small contained leakage at the proximal gastric tube just below the anastomosis site that was confirmed by the barium swallow test and endoscopy; however, it was managed successfully with conservative treatment. Consequently, stricture of the anastomosis site developed in one patient, and his hospital stay was 54 days. Another four patients complained of dysphagia after surgery. Endoscopy showed anastomotic narrowing without leakage. Therefore, periodic esophageal dilatation was required. One patient developed abdominal distention, pain, and vomiting after oral intake. Imaging studies showed small bowel hernia and obstruction through laparoscopic port site, and it was successfully treated by segmental resection of the small bowel. There were 9 patients (15.5%) with laryngeal nerve injury, 7 of whom had transient hoarseness, and 2 patients underwent vocal cord fixation. Two patients (3.4%) developed transient atrial fibrillation. Routine endoscopy was performed 3 months after surgery and the level of anastomosis was recorded in the 55 patients. The anastomotic site was identified at 22.3±1.8 cm (mean) from the incisor.

**Table 3:** Morbidity and mortality of a High Intrathoracic Anastomosis under Thoracoscopy.

Complications	Number (%)
Transfusion	2 (3.4)
Pneumonia	1 (1.7)
Empyema	None
Chylothorax	None
Atrial fibrillation	2 (3.4)
Vocal cord injury	9 (15.5)
Prolonged air leakage	None
Anastomotic leakage	3 (5.2)
Stricture	5 (8.6)
Bowel obstruction	1 (1.7)
Death	1 (1.7)

### Recurrence during F/U period

The median overall survival was 24 months (range 3–53 months). Two patients died during the follow-up period. One patient died of cerebral infarction one year after surgery that was not related to the cancer. The other patient died of pneumonia during adjuvant treatment. One patient received radiation treatment because of local recurrence at the anastomotic site 25 months after surgery without metastasis, and there was no evidence of recurrence from 12 months post-radiation treatment. Nine patients showed distant metastasis (Table 4), and the median disease-free survival time was 20 months (range 3–53 months).

**Table 4:** Follow up period for oncologic results in 57 patients.

	Number (%)
Death	2 (3.5)
Cerebral infarction	1 (1.8)
Pneumonia during adjuvant treatment	1 (1.8)
Local recurrence at the anastomosis	1 (1.8)
Distant metastasis	9 (15.8)
Supraclavicular lymph node	2 (3.5)
Brain	1 (1.8)
Bone	2 (3.5)
Liver	4 (7.0)

## Discussion

In recent years, with the improvement of endoscopic skills and instruments, many centers have adopted MIE and reported favorable postoperative surgical outcomes, including less postoperative pain, a shorter hospital stay, and fewer complications than open esophagectomy [1]. The Ivor Lewis procedure was considered the standard treatment for lower esophageal cancer. It has been reported to be preferable to the McKeown operation or trans-hiatal approach. It allows complete mediastinal lymph node dissection and has a lower incidence of postoperative anastomotic leakage, stricture and nerve injury [3]. Thus, we have adopted minimal invasive esophagectomy and thoracoscopic intrathoracic anastomosis technique since 2010 for mid- and lower ESCC.

When circular anastomosis is used, insertion of an EEA body in the stomach graft is a crucial procedure. Kim et al. demonstrated the extracorporeal insertion technique [7]. However, the intercostal space was too narrow for the EEA stapler. Segmental rib resection was routine in his study. However, we believed segmental rib resection produces more postoperative pain. Thus, we placed the EEA body into the stomach graft in the thoracic cavity through 6cm utility incision.

Maas et al. reviewed several different intrathoracic anastomosis techniques using thoracoscopy, although none were found to be superior to the others [8]. The high intrathoracic anastomosis in MIE could make the procedure more troublesome. It requires more radical dissection of the esophagus, possibly leading to more complications, including nerve injury stricture and leakage and only circular stapler anastomosis could be performed in more proximal intrathoracic regions. Furthermore, anvil placement through esophageal opening is also troublesome because handling of anvil holder is limited through the working port. Anastomosis is also difficult because of obstructive view at the apex of chest and improper angulation of circular stapler body curvature.

In our study, the level of anastomosis from upper incisor was 22.3cm. It was relatively lower level of anastomosis compared with previous study. In the literature by Park et al [9], their level of anastomosis was 20.9cm from upper incision in the high intrathoracic anastomosis. We thought the anastomosis was performed slantingly so it might have a margin of error range.

Mediastinal lymph node dissection is the major procedure in esophageal cancer surgery. Thoracoscopy enables radical lymph node dissection, including that of both recurrent laryngeal nerve lymph nodes because of the magnified view.

Shen et al. reported the results of radical lymph node dissection with minimally invasive esophagectomy [10] and found that 9.2% of patients showed hoarseness after surgery. Luketich et al. [1, 4] showed that the nerve injury rate in MIE-cervical anastomosis was significant higher with compared with MIE-intrathoracic anastomosis (8% vs. 1%). Our study showed that nine patients (15.5%) developed hoarseness due to nearby dissection of recurrent laryngeal lymph nodes, and seven patients recovered spontaneously. Nerve paralysis was relatively high compared with that in previous studies using Ivor Lewis esophagectomy. However, permanent damage is relatively low (3.4%). This result may have been obtained due to the performance of radical dissection of upper thoracic esophagus and aggressive lymph node dissection along the recurrent laryngeal nerve because recurrent laryngeal lymph node metastasis is a significant prognostic factor in ESCC [11].

One study revealed early recovery with minimally invasive Ivor Lewis esophagectomy through left lateral decubitus position compared with the open procedure [12, 13]. In particular, pulmonary complications are significantly lower with MIE. In our experience, ward ambulation, deep breathing, and active coughing are possible on the immediate postoperative day, thus reducing pulmonary complications.

Hsu et al. reported the clinical outcomes after MIE and cervical anastomosis [14]. The overall survival of the patients with stage I and II disease was similar to that of our group. This study is a single-arm and retrospective study with selection bias. Additionally, the follow-up period (median, 24 months) was not sufficiently long to enable clarification of the oncologic results. However, the oncologic results and peri-operative and short-term oncologic outcomes were similar to those of previous studies in the literature ([14–16], Table 5).

**Table 5:** Comparison data form literature.

	Our study.	Palazzo et al.	ECOG study	Hsu PK et al.
Number	58	104	104	66
Age (m or SD)	64.3±9	62.3 (31–86)	65 (36–83)	58.8 ±10.4
Complete resection(%)	58 (100%)	101 (97.1)	99 (96.1%)	64 (97%)
Operation time (m or SD)	371.8±51.6		330	510.9 ± 121.3
Number of LN (m or SD or median)	31±11.7	21 (3–57)	19 (2–55)	28.3 ± 16.6
Stage I (%)	28 (48.3)	72 (69.2)	40 (38.5)	16 (24.2)
Leakage (%)	3 (5.2)	21 (20.2)	9 (8.6)	18 (27.3)
Pneumonia (%)	1 (1.7)	7 (6.7)	4 (3.8)	7 (10.6)
30 day death (%)	1 (1.7)	2 (1.9)		5 (7.6)
F/U period	24	29.3	35.8	23.0 ±13.2
Recurrence (%)	10 (17.5)	21 (21)	29 (27.9)	20 (32.8)

ECOG: the Eastern Cooperative Oncology Group. F/U period: Follow up period.

As a conclusion, in mid- to low ESCC, MIE-intrathoracic anastomosis is feasible and safe; however, long-term follow-up is required, and a comparison of oncologic outcomes with the outcomes associated with MIE cervical anastomosis is required.

## Supporting Information

S1 Data Excel spreadsheet of patients’ data. (XLSX)
